# Tumor-Associated Macrophages and Mast Cells Positive to Tryptase Are Correlated with Angiogenesis in Surgically-Treated Gastric Cancer Patients

**DOI:** 10.3390/ijms19041176

**Published:** 2018-04-12

**Authors:** Giuseppe Sammarco, Cosmo Damiano Gadaleta, Valeria Zuccalà, Emre Albayrak, Rosa Patruno, Pietro Milella, Rosario Sacco, Michele Ammendola, Girolamo Ranieri

**Affiliations:** 1 Department of Medical and Surgical Sciences, Clinical Surgery Unit, University “Magna Graecia” Medical School, Viale Europa, Germaneto, 88100 Catanzaro, Italy; sammarco@unicz.it; 2 Interventional Oncology Unit with Integrated Section of Translational Medical Oncology National Cancer Research Centre, Istituto Tumori “Giovanni Paolo II”, viale Orazio Flacco 65, 70124 Bari, Italy; c.gadaleta@oncologico.bari.it; 3 Pathology Unit, “Pugliese-Ciaccio” Hospital, Viale Pio X, 88100 Catanzaro, Italy; valezy@libero.it; 4 Department of Medical Biochemistry, Gulhane Medical Faculty, Health Science University, Ankara 06010, Turkey; 61emrealbayrak@gmail.com; 5 Chair of Pathology, Veterinary Medical School, University “Aldo Moro” of Bari, Via Casamassima, 70010 Bari, Italy; rosavet@libero.it; 6 Statistic and Epidemiology Unit, National Cancer Research Centre, Istituto Tumori “Giovanni Paolo II”, viale Orazio Flacco 65, 70124 Bari, Italy; p.milella@oncologico.bari.it; 7 Department of Medical and Surgical Sciences, Clinical Surgery Unit, University “Magna Graecia” Medical School, Viale Europa, Germaneto, 88100 Catanzaro, Italy; sacco@unicz.it; 8 Department of Medical and Surgical Sciences, Clinical Surgery Unit, University “Magna Graecia” Medical School, Viale Europa, Germaneto, 88100 Catanzaro, Italy; 9 Surgery Unit, National Cancer Research Centre Istituto Tumori ‘‘Giovanni Paolo II’’, 70124 Bari, Italy; 10 Interventional Oncology Unit with Integrated Section of Translational Medical Oncology, National Cancer Research Centre, Istituto Tumori “Giovanni Paolo II”, viale Orazio Flacco 65, 70124 Bari, Italy; giroran@tiscalinet.it

**Keywords:** angiogenesis, microvascular density, mast cells, tryptase, macrophages, gastric cancer

## Abstract

Mast cells and macrophages can play a role in tumor angiogenesis by stimulating microvascular density (MVD). The density of mast cells positive to tryptase (MCDPT), tumor-associated macrophages (TAMs), and MVD were evaluated in a series of 86 gastric cancer (GC) tissue samples from patients who had undergone potential curative surgery. MCDPT, TAMs, and MVD were assessed in tumor tissue (TT) and in adjacent normal tissue (ANT) by immunohistochemistry and image analysis. Each of the above parameters was correlated with the others and, in particular for TT, with important clinico-pathological features. In TT, a significant correlation between MCDPT, TAMs, and MVD was found by Pearson *t*-test analysis (*p* ranged from 0.01 to 0.02). No correlation to the clinico-pathological features was found. A significant difference in terms of mean MCDPT, TAMs, and MVD between TT and ANT was found (*p* ranged from 0.001 to 0.002). Obtained data suggest MCDPT, TAMs, and MVD increased from ANT to TT. Interestingly, MCDPT and TAMs are linked in the tumor microenvironment and they play a role in GC angiogenesis in a synergistic manner. The assessment of the combination of MCDPT and TAMs could represent a surrogate marker of angiogenesis and could be evaluated as a target of novel anti-angiogenic therapies in GC patients.

## 1. Introduction

Mast cells (MCs) can play a role in tumor angiogenesis and their involvement has been found in spontaneous animal tumor models and human malignancies [1,2,3]. MCs are able to secrete classical pro-angiogenic factors, such as vascular endothelial growth factor (VEGF), fibroblast growth factor-2 (FGF-2), and thymidine phosphorylase (TP), and they are also capable to secrete non-classical pro-angiogenetic factors. [4,5]. Among these, tryptase is the most abundant factor stored in MC secretory granules and it can be degranulated in several ways, for example, C-Kit receptor activation. Published data has indicated that tryptase stimulates endothelial cell (EC) proliferation in a matrigel assay and induces microvascular proliferation in the chick embryo chorioallantoic membrane. In the last experimental model, microvascular proliferation was suppressed by gabexate, a tryptase inhibitor. It is well demonstrated that tryptase binds protease-activated receptor-2 (PAR-2) expressed on ECs, stimulating microvascular formation [6,7,8,9,10,11,12,13,14,15,16,17,18,19,20,21,22,23,24,25,26,27,28,29,30,31,32,33,34,35,36]. Among the stromal cells homing to tumor microenvironments, macrophages (Ms) play several important roles [37]. In tumor microenvironments there are two types of Ms, Ms1 and Ms2 [37]. In particular, Ms1 are involved in inflammation and immune activity against tumor [38]. Ms2 are identified as tumor-associated macrophages (TAMs) and they play a role as pro-angiogenic cells [39,40]. It has been observed that Ms synthesize well known pro-angiogenic factors, particularly VEGF, TP, FGF-2, tumor necrosis factor-α (TNF-α), and interleukins (IL-1, -6, and -8). After exocytosis from Ms2, the above factors stimulate ECs towards proliferation, differentiation, survival, migration, and vascular permeability, thus leading to new microvessel formation [41,42]. Ms also induce angiogenesis by producing and releasing metalloproteinase-9 (MMP-9) that is able to degrade the extracellular matrix and then cause the release of VEGF [43,44].

In this context, very little data have been published on the relationship among MCs density positive to tryptase (MCDPT), TAMs, and microvascular density (MVD) in primary gastric cancer (GC) tissue [45,46,47,48,49,50].

In this pilot study, we analyzed MCDPT, TAMs and MVD in tumor tissue (TT) and in adjacent normal tissue (ANT) by immunohistochemistry and image analysis from 86 GC patients who had undergone radical surgery. The correlation between the studied parameters and the main clinico-pathological features has also been determined.

## 2. Results

**Figure 1:**
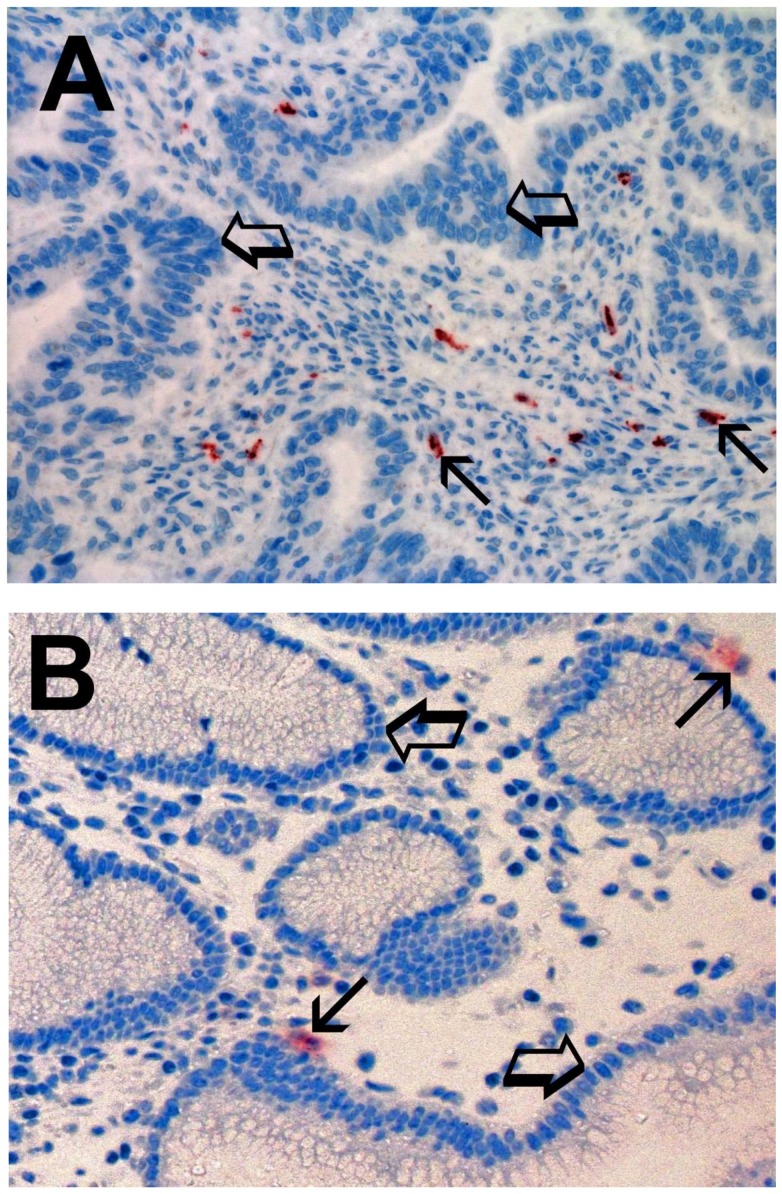
(**A**) High MCD in primary gastric cancer tissue section immunostained with the anti-tryptase antibody. Small arrows indicate single red stained mast cells. Big arrows indicate tumoral epithelium. Magnification ×400 (0.19 mm^2^ area). (**B**) Low MCD in adjacent normal tissue section immunostained with the anti-tryptase antibody. Small arrows indicate single red stained mast cells. Big arrows indicate normal gastric epithelium. Magnification ×400 (0.19 mm^2^ area).

Immunostained MCs appear as ovoidal-elongated cells with a red marked cytoplasm and a blue stained nucleus. MCs were scattered in the stromal microenvironment and it was evident that MCDPT were increased in TT vs. ANT (Figure 1A vs. Figure 1B). For each serial section of TT and ANT, the five most immunostained areas (hot spots) were selected and MCDPT was counted in each hot spot and then the mean for each section and in the global series was calculated (Table 1).

**Figure 2:**
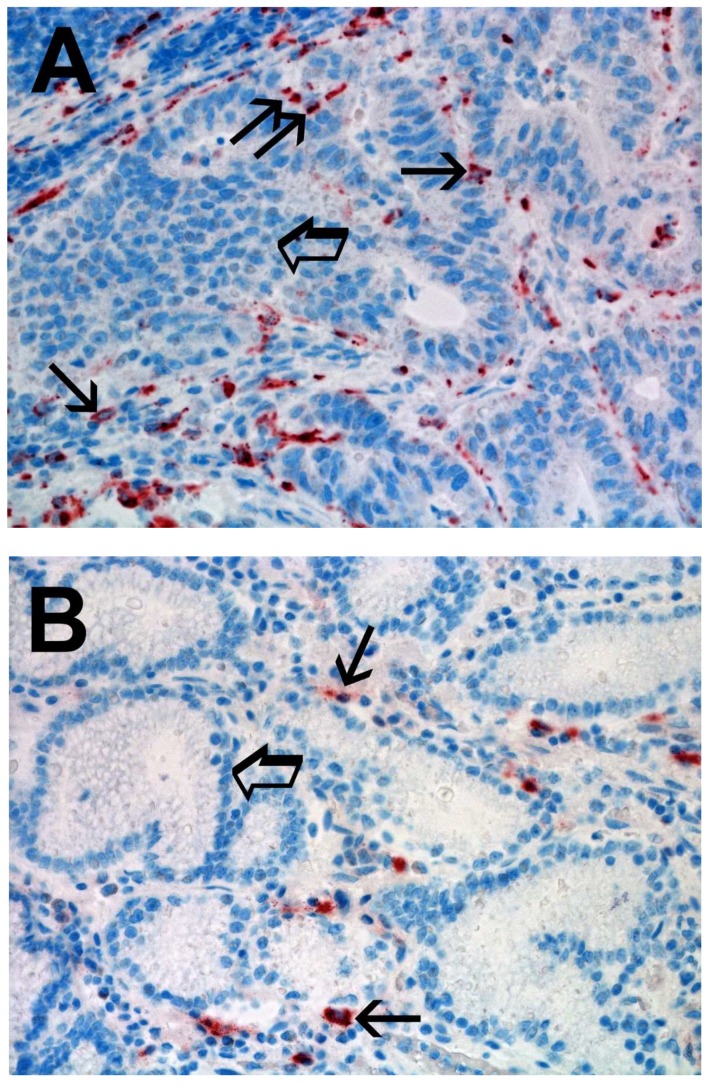
(**A**) High TAMs count in primary gastric cancer tissue section immunostained with the anti-CD68 antibody. Small arrows indicate single red stained macrophages. Big arrow indicates tumoral epithelium. Magnification ×400 (0.19 mm^2^ area). (**B**) Low TAMs count in adjacent normal tissue section immunostained with the anti-CD68 antibody. Small arrows indicate single red stained macrophages. Big arrow indicates normal gastric epithelium. Magnification ×400 (0.19 mm^2^ area).

Immunostained TAMs were identified as red marked cells with a blue nucleus. They were more pleomorphic and more abundant compared to MCs. With the image analysis system, two main shapes of Ms were seen: the first with an irregularly defined cytoplasm and a waving plasmalemma (like ameboid cell) with central nucleus (Figure 2A, single small arrow in the upper part); the second with an elongate cytoplasm and a well evident central nucleus (Figure 2A, single small arrow in the lower part). A higher count of TAMs was detected in TT vs. ANT (Figure 2A vs. Figure 2B). For each serial section of TT and ANT, the five most immunostained areas (hot spots) were selected and MCDPT were counted in each hot spot and then the mean for each section and in the global series was calculated (Table 1).

**Figure 3:**
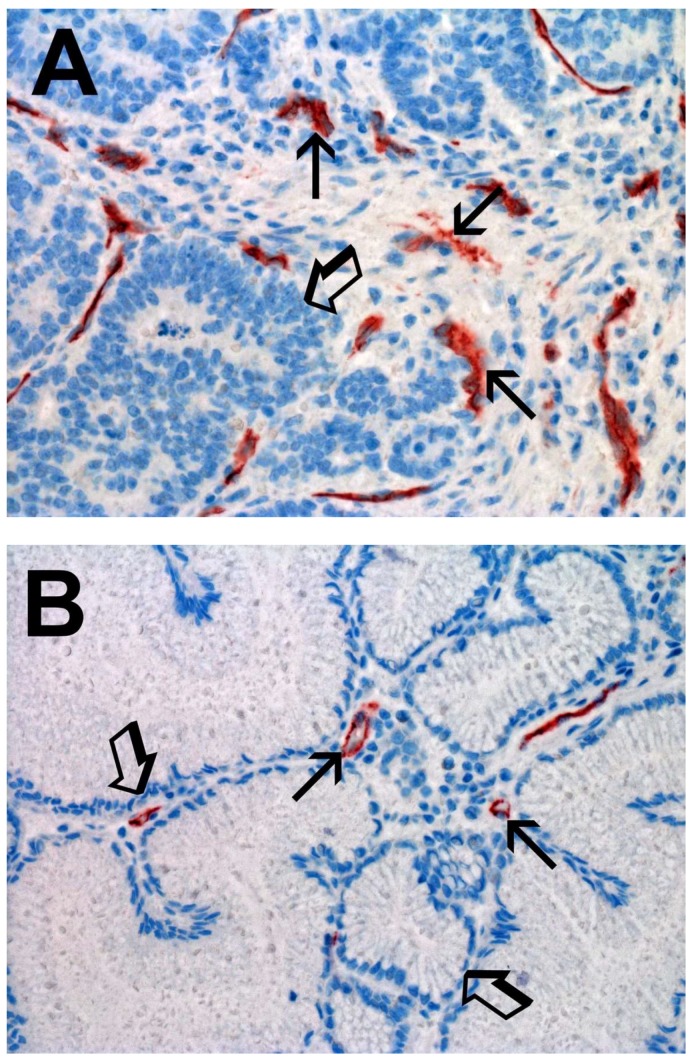
(**A**) High MVD in primary gastric cancer tissue section immunostained with the anti-CD31 antibody. Small arrows indicate single red stained microvessels. Small arrows indicate single red stained macrophages. Big arrow indicates tumoral epithelium. Magnification ×400 (0.19 mm^2^ area). (**B**) Low MVD in adjacent normal tissue section immunostained with the anti-CD31 antibody. Small arrows indicate single red stained microvessels. Big arrow indicates normal gastric epithelium. Magnification ×400 (0.19 mm^2^ area).

For MVD, each red immunostained cell was considered a single microvessel and the diameter of the microvessel and the number lumen and red blood cells present in the lumen were considered when identifying a microvessel. Microvessels were scattered within stromal tissue. The blue nuclei of endothelial cells were often evident and higher MVD was observed in TT vs. ANT (Figure 3A vs. Figure 3B).

**Figure 4:**
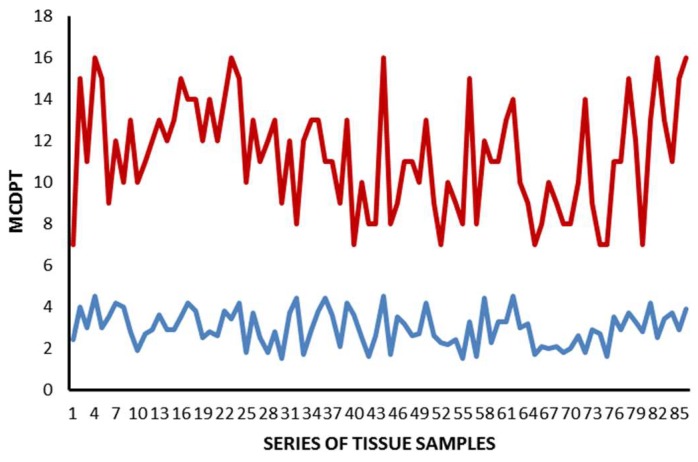
Actual value regarding MCDPT in TT (red) and ANT (blue).

**Figure 5:**
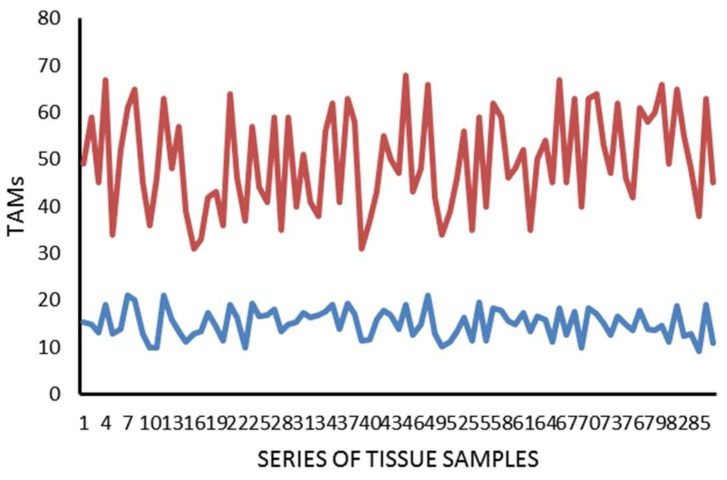
Actual value regarding TAMs in TT (red) and ANT (blue).

**Figure 6:**
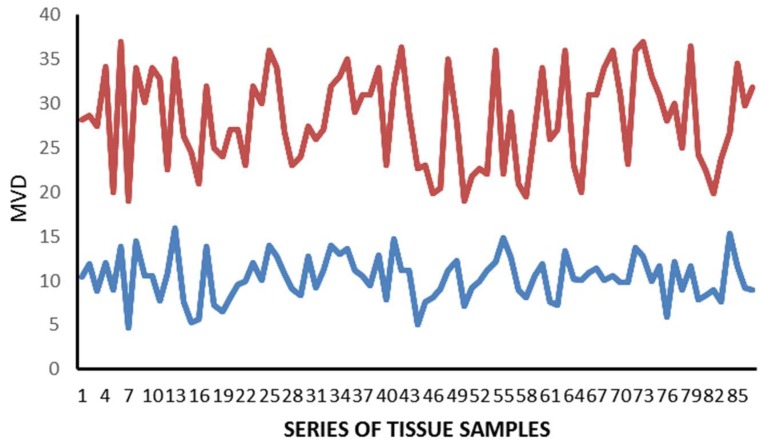
Actual value regarding MVD in TT (red) and ANT (blue).

**Figure 7:**
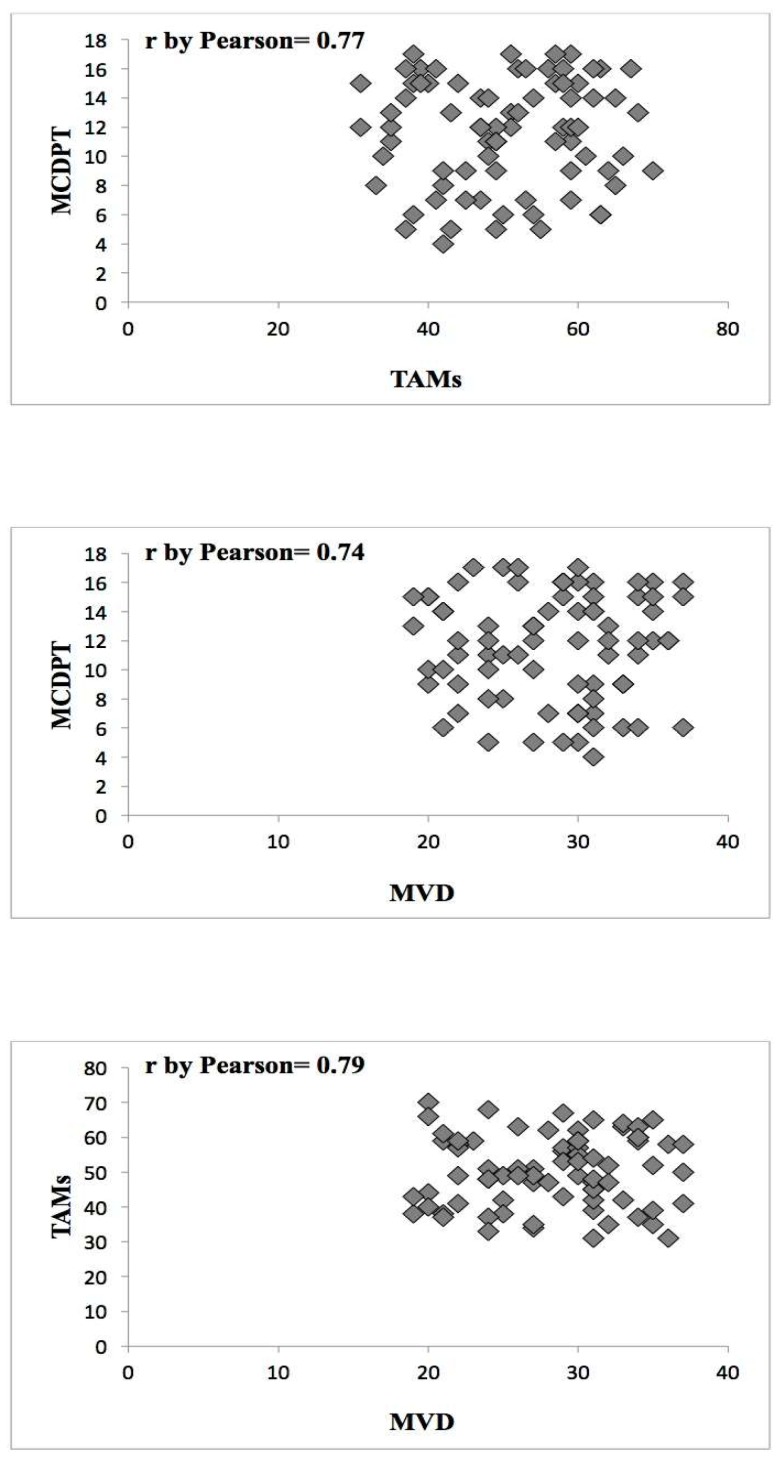
Correlation between MCDPT and TAMs (*r* = 0.77, *p* = 0.02), MCDPT and MVD (*r* = 0.74, *p* = 0.02), and TAMS and MVD (*r* = 0.79, *p* = 0.01) in gastric cancer tumor tissue.

The mean value ± standard deviation (SD) regarding MCDPT, TAMs, and MVD in TT and ANT was 11.38 ± 4.32, 49.17 ± 17.56, 28.12 ± 8.98 and 2.98 ± 1.45, 15.34 ± 6.21, 10.39 ± 5.62, respectively, and these differences were significant (*p* ranged from 0.001 to 0.002; Table 1). The actual values of MCDPT, TAMs, and MVD in TT and ANT are shown in the histograms (Figure 4, Figure 5 and Figure 6) respectively. Interestingly, in TT, a higher MVD was associated with higher MCDPT and TAMs, thus supporting the role of these stromal cells in neovascularization. From a statistical point of view, significant correlations between MCDPT and TAMs (*r =* 0.77, *p* = 0.02), MCDPT and MVD (*r =* 0.74, *p* = 0.02), and TAMS and MVD (*r* = 0.79, *p* = 0.01) (Figure 7) were shown. With special regard to TT, no correlation between MCDPT, TAMs, MVD, and the main clinic-pathological features was found (Table 2).

## 3. Discussion

Substantial data has indicated the key role of angiogenesis in GC development and progression, but little data related to the role of both MCDPT and TAMs in GC angiogenesis have been published [40,51,52]. Tumor angiogenesis is the process of new blood vessel formation from the pre-existing vascular network. This process takes place in the tumor microenvironment where stromal cells, particularly macrophages and mast cells, induce microvessel formation by means of pro-angiogenic factors [53,54,55,56]. In the stromal microenvironment, MCs and TAMs can be attracted by the pro-inflammatory cytokines secreted from tumor cells and other inflammatory cells. A lot of research has indicated that either MCDPT and TAMs are correlated with MVD degree in animal and human malignancies [6,7,8,9,10,11,12,13,14,15,16,17,18,19,20,21,22,23,24,25,26,27,28,29,30,31,32,33,34,35,36,39,40,41,42], but little data have been reported concerning the concomitant evaluation of MCDPT and TAMs and their correlation with tumor angiogenesis in terms of MVD.

With special regard to MCs, Mukherjee et al. [57] assessed MC density in tissue from patients affected by gastric ulcers and by GC. Data from this research group were obtained utilizing the histochemical stain of toluidine blue to identify and count MC density. Results indicated that MC density increased in benign gastric ulcers and in cancers compared to control tissue, and furthermore that MC density correlated with angiogenesis.

Ribatti et al. [47] studied TT from GC patients using immunohistochemistry with anti-tryptase and anti-chymase antibodies to stain MCs. This study showed a correlation between MVD and tryptase and chymase-positive MCs, demonstrating the role of MCs in neovascularization.

Recently, we published data indicating that MCDPT may induce angiogenesis in bone metastases from gastric cancer patients, suggesting that MCDPT is able to stimulate angiogenesis in metastatic tumor sites [31].

In the tumor microenvironment, MCs can be activated in different ways, including c-kit receptor stimulation by its ligand, stem cell factors, the IgE-dependent mechanism mediated by T lymphocyte–MC interaction, and TLR activation by other microenvironmental stimuli [58,59]. After activation, intensive or piecemeal degranulation of secretory granules occurs and MC-derived tryptase is released into the tumor microenvironment.

In bench experimental matrigel assays and in chick embryo chorioallantoic membrane assays, tryptase induces EC proliferation and neovascularization which was suppressed by tryptase inhibitors. Tryptase is an agonist of PAR-2 on vascular ECs that induces their proliferation. Following PAR-2 stimulation, the MAPK pathway is stimulated, leading to proliferation and angiogenesis [6,7,8,9,10,11,12,13,14,15,16,17,18,19,20,21,22,23,24,25,26,27,28,29,30,31,32,33,34,35,36].

Ms synthesize and release numerous pro-angiogenic substances, such as VEGF, TP, FGF-2, TNF-α, and IL-1, -6, and -8, which stimulate EC proliferation, migration, and differentiation. Ms can also indirectly stimulate angiogenesis by producing MMP-9 that degrades the extracellular matrix and releases VEGF [37,38,39,40,41,42,43,44].

MCDPT and TAMs are linked via Toll-like receptors (TLRs). Recent published pilot data from our group demonstrated that MCDPT and TAMs paralleled each other in colon cancer tissue and they increase with increased angiogenesis. TLRs are a family of membrane-spanning, non-catalytic receptors expressed in immune stromal cells including TAMs and MCs [40,41,59].

After TLR activation, intracellular signaling is conducted in two different biological ways.

Firstly, the MyD88-dependent pathway is activated, leading to the activation of nuclear factor κ (NF-κB) and mitogen-activated protein kinase (MAPK). Secondly, the TIR-domain-containing adapter-inducing interferon-β dependent pathway is activated, leading to the activation of serine/threonine-protein kinase-1 and receptor-interacting serine/threonine-protein kinase 1 [60,61]. The final result of these intracellular signal cascades is secretion of several pro-angiogenic factors, such as VEGF, TP, FGF-2, TNF-α, IL-1, -6, -8, and tryptase [62].

Increased expression of TLRs has been demonstrated in tumor cells, tissues, and tumor cell lines. TLR4 has been observed as over-expressed in human and mouse colorectal neoplasia, and on the other hand, TLR4-deficient mice are refractory to colon carcinogenesis, indicating that the higher TLR presence on tumor cells induces tumor development directly or indirectly by mean of angiogenesis [61].

According to these biological backgrounds, our results suggested that the increment of both MCDPT and TAMs were correlated with increased MVD. Our results also demonstrated that MCDPT, TAMs, and MVD are much higher in TT compared to ANT, supporting the theory that the above cell types play a role in tumor development and angiogenesis.

To the best of our knowledge, no other study has been published regarding the concomitant assessment of both MCDPT and TAMs and neovascularization in TT and ANT from GC patients. We propose that the coupled MCDPT and TAMs could be considered as a surrogate biomarker of the degree of GC angiogenesis [50,63,64,65]. From a therapeutic point of view, the data obtained support a novel possibility to inhibit GC angiogenesis by targeting the coupled TAMs and MCDPTs by mean of several agents (e.g., trabectedin, peptide M2, PLX3397, STI571, AB1010). Finally, available tryptase inhibitors such as Gabexate or Nafamostat mesilate may be tested in future clinical trials as an innovative anti-angiogenic strategy [66,67,68,69,70].

## 4. Materials and Methods

### 4.1. Study Population

Eighty-six GC patients diagnosed by preoperative gastric endoscopy were selected to undergo potential curative surgery resection. The surgical techniques were open total and sub-total gastrectomy with D2 lymph node dissection. Selected cases were staged as T_2–3_N_2–3_M_0_ (by AJCC for Gastric Cancer 7th Edition) according to the American Joint Committee on Cancer 7th edition (AJCC-TNM) classification [71,72,73,74]. Clinical staging was performed by computed tomography (CT) of the thorax, abdomen, and pelvis. All enrolled patients had adenocarcinomas. The main clinico-pathological characteristics of the patients are reported in Table 2. The research was developed according to the Declaration of Helsinki, and the study was approved by the Ethics Committee of the “Mater Domini” Hospital, “Magna Graecia” University, Catanzaro (No. 242; 22 December 2016). Signed consent from each patient was obtained.

### 4.2. Immunohistochemistry

MCDPT, TAMs, and MVD were detected by immunohistochemistry using a three-layer biotin-avidin-peroxidase system [75]. Briefly, 6-μm-thick serial sections of formalin-fixed and paraffin-embedded TT and ANT were cut. Obtained slides were processed with a microwave oven at 500 W for 10 min, and then the endogenous peroxidase enzyme was inhibited with 3% hydrogen peroxide solution. Subsequently, slides were posted with the following primary antibodies: anti-tryptase (clone AA1; Dako, Glostrup, Denmark) diluted 1:100 for 1 h at room temperature (for MCs identification), anti-CD68 (clone KP1; Dako, Glostrup, Denmark) diluted 1:100 for 1 h at room temperature as the TAMs marker, anti-CD31 antibody (QB-END 10; Bio-Optica Milan, Milan, Italy) diluted 1:50 for 1 h at room temperature as a pan-endothelial marker. The immunoreactivity was detected by employing a biotinylated secondary antibody, and the avidin–biotin peroxidase complex yielded a red chromogen (LPS, K0640, Dako, Glostrup, Denmark). Cell nuclei were stained with Gill’s haematoxylin No. 2 (Polysciences, Warrington, PA, USA). No primary antibody was posted in negative controls.

### 4.3. Morphometrical Assay

Light microscopy integrated with an image analysis system (AXIO, Scope A1, ZEISS, Germany) was utilized [75]. For each serial section of TT and ANT, the five most immunostained areas (hot spots) were selected at low magnification. Next MCDPT, TAMs, and MVD were assessed at ×400 magnification (0.19 mm^2^ area) in the five identified hot spots areas for each serial section, respectively (Figure 1A,B, Figure 2A,B and Figure 3A,B). With special reference to MCDPT and TAMS, each immunostained cell was considered in their count. Furthermore, MVD was detected by counting single red-brown stained endothelial cells, endothelial cell clusters, and microvessels, clearly separated from adjacent microvessels.

### 4.4. Statistical Analysis

Mean values for each section and in the global series was obtained for all studied parameters in both TT and ANT groups. The difference between groups was measured by Student’s *t*-test. Mean values ± 1 Standard Deviation (SD) of all the evaluated tissue parameters are reported in Table 1.

Correlations between MCDPT, TAMs, and MVD were calculated using Pearson’s (*r*) analysis (Figure 4). Correlations among all the analyzed parameters and the main clinico-pathological features listed in Table 2 were performed by the χ-square test (χ^2^). All analyses were considered statistically significant with a *p* < 0.05. Statistical analyses were performed with the SPSS statistical software package (SPSS, Inc., Chicago, IL, USA).

## Figures and Tables

**Table 1:** Mast cells density positive to tryptase (MCDPT), tumor-associated macrophages (TAMs), and microvascular density (MVD) means ± standard deviations as a function of gastric cancer (GC) tumor tissue (TT) and adjacent normal tissue (ANT), respectively.

Tissue	MCDPT ×400 Magnification (0.19 mm^2^ Area)	TAMs ×400 Magnification (0.19 mm^2^ Area)	MVD ×400 Magnification (0.19 mm^2^ Area)
TT	11.38 ± 4.32 ^a^	49.17 ± 17.56 ^a^	28.12 ± 8.98 ^a^
ANT	2.98 ± 1.45 ^a^	15.34 ± 6.21 ^a^	10,39 ± 5.62 ^a^
*t*-test	*p* = 0.001	*p* = 0.001	*p* = 0.002

^a^ Mean ± 1 standard deviation.

**Table 2:** MCDPT, TAMs, and MVD as functions of clinico-pathological characteristics in a series of 86 gastric cancer patients.

Variable	No. of Patients	No. of Tumors with High MCDPT ^a^ (%)	No. of Tumors with High MVD ^b^ (%)	No. of Tumors with High TAMs ^c^ (%)
Age years	86			
≤65	35	19 (54)	18 (51)	17 (49)
≥65	51	25 (49)	24 (47)	26 (51)
Gender				
►Male	52	25 (48)	24 (46)	27 (52)
►Female	34	17 (50)	18 (53)	16 (47)
Tumor site				
►Cardia, Lesser and Greater curvature	31	17 (55)	15 (48)	16 (52)
►Body and fundus	26	13 (50)	14 (54)	13 (50)
►Pyloric area	29	14 (48)	15 (52)	14 (48)
TNM by AJCC Stage				
►T_2–3_N_2_M_0_	52	24 (46)	28 (54)	27 (52)
►T_2–3_N_3_M_0_	34	18 (53)	16 (47)	17 (50)
Tumor type by Lauren Classification				
►Intestinal type	55	26 (47)	27 (49)	29 (53)
►Diffuse type	31	15 (48)	17 (55)	16 (52)
Histologic grade				
►G1–G2	42	22 (52)	21 (50)	23 (55)
►G3	44	24 (54)	21 (47)	20 (45)

^a^ Median cut-off value: 11 cells per 400 field; ^b^ Median cut-off value: 29 microvessels per 400 field; ^c^ Median cut-off value: 48 cells per 400 field.

## References

[1] Bhattacharyya S.P., Drucker I., Reshef T., Kirshenbaum A.S., Metcalfe D.D., Mekori Y.A. (1998). Activated T lymphocytes induce degranulation and cytokine production by human mast cells following cell-to-cell contact. J. Leukoc. Biol..

[2] Marech I., Ammendola M., Sacco R., Sammarco G., Zuccalà V., Zizzo N., Leporini C., Luposella M., Patruno R., Filippelli G. (2016). Tumour-associated macrophages correlate with microvascular bed extension in colorectal cancer patients. J. Cell. Mol. Med..

[3] Patruno R., Marech I., Zizzo N., Ammendola M., Nardulli P., Gadaleta C., Introna M., Capriuolo G., Rubini R.A., Ribatti D. (2014). c-Kit expression, angiogenesis, and grading in canine mast cell tumour: A unique model to study c-Kit driven human malignancies. Biomed. Res. Int..

[4] Wasiuk A., de Vries V.C., Hartmann K., Roers A., Noelle R.J. (2009). Mast cells as regulators of adaptive immunity to tumours. Clin. Exp. Immunol..

[5] Norrby K. (2002). Mast cells and angiogenesis. APMIS.

[6] Ribatti D., Ranieri G. (2015). Tryptase, a novel angiogenic factor stored in mast cell granules. Exp. Cell Res..

[7] Marone G., Varricchi G., Loffredo S., Granata F. (2015). Mast cells and basophils in inflammatory and tumor angiogenesis and lymphangiogenesis. Eur. J. Pharmacol..

[8] Zhang X., Wang W., Mize G.J., Takayama T.K., True L.D., Vessella R.L. (2013). Protease-activated receptor 2 signaling up regulates angiogenic growth factors in renal cell carcinoma. Exp. Mol. Pathol..

[9] Rasmussen J.G., Riis S.E., Frobert O., Yang S., Kastrup J., Zachar V., Simonsen U., Fink T. (2012). Activation of protease-activated receptor 2 induces VEGF independently of HIF-1. PLoS ONE.

[10] Chang L.H., Pan S.L., Lai C.Y., Tsai A.C., Teng C.M. (2013). Activated PAR-2 regulates pancreatic cancer progression through ILK/HIF-α-induced TGF-α expression and MEK/VEGF-A-mediated angiogenesis. Am. J. Pathol..

[11] Ammendola M., Sacco R., Sammarco G., Piardi T., Zuccalà V., Patruno R., Zullo A., Zizzo N., Nardo B., Marech I. (2016). Mast Cells positive to tryptase, endothelial cells positive to protease-activated receptor-2, and microvascular density correlate among themselves in hepatocellular carcinoma patients who have undergone surgery. OncoTargets Ther..

[12] Ammendola M., Sacco R., Marech I., Sammarco G., Zuccalà V., Luposella M., Patruno R., Giordano M., Ruggieri E., Zizzo N. (2015). Microvascular density and endothelial area correlate with Ki-67 proliferative index in surgically-treated pancreatic ductal adenocarcinoma patients. Oncol Lett..

[13] Ammendola M., Sacco R., Sammarco G., Luposella M., Patruno R., Gadaleta C.D., de Sarro G., Ranieri G. (2016). Mast Cell-Targeted Strategies in Cancer Therapy. Transfus. Med. Hemother..

[14] Marech I., Ammendola M., Sacco R., Capriuolo G.S., Patruno R., Rubini R., Luposella M., Zuccalà V., Savino E., Gadaleta C.D. (2014). Serum tryptase, mast cells positive to tryptase and microvascular density evaluation in early breast cancer patients: Possible translational significance. BMC Cancer.

[15] Marech I., Ammendola M., Gadaleta C., Zizzo N., Oakley C., Gadaleta C.D., Ranieri G. (2014). Possible biological and translational significance of mast cells density in colorectal cancer. World J. Gastroenterol..

[16] Ammendola M., Sacco R., Sammarco G., Donato G., Montemurro S., Ruggieri E., Patruno R., Marech I., Cariello M., Vacca A. (2014). Correlation between serum tryptase, mast cells positive to tryptase and microvascular density in colo-rectal cancer patients: Possible biological-clinical significance. PLoS ONE.

[17] Ammendola M., Sacco R., Sammarco G., Donato G., Zuccalà V., Romano R., Luposella M., Patruno R., Vallicelli C., Verdecchia G.M. (2013). Mast Cells Positive to Tryptase and c-Kit Receptor Expressing Cells Correlates with Angiogenesis in Gastric Cancer Patients Surgically Treated. Gastroenterol. Res. Pract..

[18] Ammendola M., Sacco R., Donato G., Zuccalà V., Russo E., Luposella M., Vescio G., Rizzuto A., Patruno R., De Sarro G. (2013). Mast cell positivity to tryptase correlates with metastatic lymph nodes in gastrointestinal cancer patients treated surgically. Oncology.

[19] Marech I., Leporini C., Ammendola M., Porcelli M., Gadaleta C.D., Russo E., de Sarro G., Ranieri G. (2015). Classical and non classical proangiogenic factors as a target of antiangiogenic therapy in tumor microenvironment. Cancer Lett..

[20] Ribatti D., Ranieri G., Nico B., Benagiano V., Crivellato E. (2011). Tryptase and chymase are angiogenic in vivo in the chorioallantoic membrane assay. Int. J. Dev. Biol..

[21] Blair R.J., Meng H., Marchese M.J., Ren S., Schwartz L.B., Tonnesen M.G., Gruber B.L. (1997). Human mast cells stimulate vascular tube formation. Tryptase is a novel, potent angiogenic factor. J. Clin. Investig..

[22] Itoh Y., Sendo T., Oishi R. (2005). Physiology and pathophysiology of proteinase-activated receptors (PARs): Role of tryptase/PAR-2 in vascular endothelial barrier function. J. Pharmacol. Sci..

[23] Rickard A., Portell C., Kell P.J., Vinson S.M., McHowat J. (2005). Protease-activated receptor stimulation activates a Ca^2+^-independent phospholipase A2 in bladder microvascular endothelial cells. Am. J. Physiol. Renal Physiol..

[24] Matej R., Mandàkovà P., Netikovà I., Pouckova P., Olejár T. (2007). Proteinase-activated receptor-2 expression in breast cancer and the role of trypsin on growth and metabolism of breast cancer cell line MDA MB-231. Physiol. Res..

[25] Morris D.R., Ding Y., Ricks T.K., Gullapalli A., Wolfe B.L., Trejo J. (2006). Protease-activated receptor-2 is essential for factor VIIa and Xa-induced signaling, migration, and invasion of breast cancer cells. Cancer Res..

[26] Ammendola M., Leporini C., Marech I., Gadaleta C.D., Scognamillo G., Sacco R., Sammarco G., de Sarro G., Russo E., Ranieri G. (2014). Targeting mast cells tryptase in tumor microenvironment: A potential antiangiogenetic strategy. BioMed Int. Res..

[27] Ammendola M., Sacco R., Sammarco G., Donato G., Zuccalà V., Luposella M., Patruno R., Marech I., Montemurro S., Zizzo N. (2014). Mast cells density positive to tryptase correlates with angiogenesis in pancreatic ductal adenocarcinoma patients having undergone surgery. Gastroenterol. Res. Pract..

[28] Donato G., Conforti F., Camastra C., Ammendola M., Donato A., Renzulli A. (2014). The role of mast cell tryptases in cardiac myxoma: Histogenesis and development of a challenging tumor. Oncol. Lett..

[29] Ammendola M., Zuccalà V., Patruno R., Russo E., Luposella M., Amorosi A., Vescio G., Sammarco G., Montemurro S., de Sarro G. (2013). Tryptase-positive mast cells and angiogenesis in keloids: A new possible post-surgical target for prevention. Updates Surg..

[30] Ranieri G., Ammendola M., Patruno R., Celano G., Zito F.A., Montemurro S., Rella A., di Lecce V., Gadaleta C.D., de Sarro G.B. (2009). Tryptase-positive mast cells correlate with angiogenesis in early breast cancer patients. Int. J. Oncol..

[31] Ammendola M., Marech I., Sammarco G., Zuccalà V., Luposella M., Zizzo N., Patruno R., Crovace A., Ruggieri E., Zito A.F. (2015). Infiltrating mast cells correlate with angiogenesis in bone metastases from gastric cancer patients. Int. J. Mol. Sci..

[32] Malfettone A., Silvestris N., Saponaro C., Ranieri G., Russo A., Caruso S., Popescu O., Simone G., Paradiso A., Mangia A. (2013). High density of tryptase-positive mast cells in human colorectal cancer: A poor prognostic factor related to protease-activated receptor 2 expression. J. Cell. Mol. Med..

[33] Soreide K., Janssen E.A., Körner H., Baak J.P. (2006). Trypsin in colorectal cancer: Molecular biological mechanisms of proliferation, invasion, and metastasis. J. Pathol..

[34] Darmoul D., Marie J.C., Devaud H., Gratio V., Laburthe M. (2001). Initiation of human colon cancer cell proliferation by trypsin acting at protease-activated receptor-2. Br. J. Cancer.

[35] Uusitalo-Jarvinen H., Kurokawa T., Mueller B.M., Andrade-Gordon P., Friedlander M., Ruf W. (2007). Role of protease activated receptor 1 and 2 signaling in hypoxia-induced angiogenesis. Arterioscler. Thromb. Vasc. Biol..

[36] Liu Y., Mueller B.M. (2006). Protease-activated receptor-2 regulates vascular endothelial growth factor expression in MDA-MB-231 cells via MAPK pathways. Biochem. Biophys. Res. Commun..

[37] Caronni N., Savino B., Bonecchi R. (2015). Myeloid cells in cancer-related inflammation. Immunobiology.

[38] Wang N., Liang H., Zen K. (2014). Molecular mechanisms that influence the macrophage M1-M2 polarization balance. Front. Immunol..

[39] Gosselin D., Link V.M., Romanoski C.E., Fonseca G.J., Eichenfield D.Z., Spann N.J., Stender J.D., Chun H.B., Garner H., Geissmann F. (2014). Environment drives selection and function of enhancers controlling tissue-specific macrophage identities. Cell.

[40] Sunderkotter C., Steinbrink K., Goebeler M., Bhardwaj R.A., Sorg C. (1994). Macrophages and angiogenesis. J. Leukoc. Biol..

[41] Chanmee T., Ontong P., Konno K., Itano N. (2014). Tumor-associated macrophages as major players in the tumor microenvironment. Cancers.

[42] Polverini P.J., Leibovich S.J. (1984). Induction of neovascularization in vivo and endothelial proliferation in vitro by tumor-associated macrophages. Lab. Investig..

[43] Polverini P.J. (1996). How the extracellular matrix and macrophages contribute to angiogenesis-dependent diseases. Eur. J. Cancer.

[44] Mantovani A. (1994). Tumor-associated macrophages in neoplastic progression: A paradigm for the in vivo function of chemokines. Lab. Investig..

[45] Yano H., Kinuta M., Tateishi H., Nakano Y., Matsui S., Monden T., Okamura J., Sakai M., Okamoto S. (1999). Mast cell infiltration around gastric cancer cells correlates with tumour angiogenesis and metastasis. Gastric Cancer.

[46] Sedda S., Marafini I., Caruso R., Pallone F., Monteleone G. (2014). Proteinase activated-receptors-associated signaling in the control of gastric cancer. World J. Gastroenterol..

[47] Ribatti D., Guidolin D., Marzullo A., Nico B., Annese T., Benagiano V., Crivellato E. (2010). Mast cells and angiogenesis in gastric carcinoma. Int. J. Exp. Pathol..

[48] Wang G.J., Wang Y.B., Li D.N., Deng B.B. (2013). Expression of protease-activated receptor-2 in human gastric stromal tumor and its clinic-pathological significance. Hepatogastroenterology.

[49] Zhang C., Gao G.R., Lv C.G., Zhang B.L., Zhang Z.L., Zhang X.F. (2012). Protease-activated receptor-2 induces expression of vascular endothelial growth factor and cyclooxygenase-2 via the mitogen-activated protein kinase pathway in gastric cancer cells. Oncol. Rep..

[50] Ammendola M., Patruno R., Sacco R., Marech I., Sammarco G., Zuccalà V., Luposella M., Zizzo N., Gadaleta C., Porcelli M. (2016). Mast cells positive to tryptase and tumour-associated macrophages correlate with angiogenesis in locally advanced colorectal cancer patients undergone to surgery. Expert Opin. Ther. Targets.

[51] Wang X., Chen X., Fang J., Yang C. (2013). Overexpression of both VEGF-A and VEGF-C in gastric cancer correlates with prognosis, and silencing of both is effective to inhibit cancer growth. Int. J. Clin. Exp. Pathol..

[52] Khazaie K., Blatner N.R., Khan M.W., Gounari F., Gounaris E., Dennis K., Bonertz A., Tsai F.N., Strouch M.J., Cheon E. (2011). The significant role of mast cells in cancer. Cancer Metast. Rev..

[53] Folkman J. (1992). The role of angiogenesis in tumor growth. Semin. Cancer Biol..

[54] Anaka T., Ishikawa H. (2013). Mast cells and inflammation-associated colorectal carcinogenesis. Semin. Immunopathol..

[55] Ribatti D., Ranieri G., Basile A., Azzariti A., Paradiso A., Vacca A. (2012). Tumor endothelial markers as a target in cancer. Expert Opin. Ther. Targets.

[56] Zhao Y., Wu K., Cai K., Zhai R., Tao K., Wang G., Wang J. (2012). Increased numbers of gastric-infiltrating mast cells and regulatory T cells are associated with tumor stage in gastric adenocarcinoma patients. Oncol. Lett..

[57] Mukherjee S., Bandyopadhyay G., Dutta C., Bhattacharya A., Karmakar R., Barui G. (2009). Evaluation of endoscopic biopsy in gastric lesions with a special reference to the significance of mast cell density. Indian J. Pathol. MicroBiol..

[58] Pimentel-Nunes P., Goncalves N., Boal-Carvalho I., Afonso L., Lopes P., Roncon-Albuquerque R., Soares J.B., Cardoso E., Henrique R., Moreira-Dias L. (2012). Decreased Toll-interacting protein and peroxisome proliferator-activated receptor gamma are associated with increased expression of Toll-like receptors in colon carcinogenesis. J. Clin. Pathol..

[59] Furuta T., Imajo-Ohmi S., Fukuda H., Kano S., Miyake K., Watanabe N. (2008). Mast cell-mediated immune responses through IgE antibody and Toll-like receptor 4 by malarial peroxiredoxin. Eur. J. Immunol..

[60] Zorn C.N., Keck S., Hendriks R.W., Leitges M., Freudenberg M.A., Huber M. (2009). Bruton’s tyrosine kinase is dispensable for the Toll-like receptor-mediated activation of mast cells. Cell. Signal..

[61] He W., Liu Q., Wang L., Chen W., Li N., Cao X. (2007). TLR4 signaling promotes immune escape of human lung cancer cells by inducing immunosuppressive cytokines and apoptosis resistance. Mol. Immunol..

[62] Wolska A., Lech-Maranda E., Robak T. (2009). Toll-like receptors and their role in carcinogenesis and anti-tumor treatment. Cell. Mol. Biol. Lett..

[63] Ammendola M., Sacco R., Vescio G., Zuccalà V., Luposella M., Patruno R., Zizzo N., Gadaleta C., Marech I., Ruggieri R. (2017). Tryptase mast cell density, protease-activated receptor-2 microvascular density, and classical microvascular density evaluation in gastric cancer patients undergoing surgery: Possible translational relevance. Therap. Adv. Gastroenterol..

[64] Ammendola M., Sacco R., Zuccalà V., Luposella M., Patruno R., Gadaleta P., Zizzo N., Gadaleta C.D., de Sarro G., Sammarco G. (2016). Mast Cells Density Positive to Tryptase Correlate with Microvascular Density in both Primary Gastric Cancer Tissue and Loco-Regional Lymph Node Metastases from Patients That Have Undergone Radical Surgery. Int. J. Mol. Sci..

[65] Ammendola M., Gadaleta C.D., Frampton A.E., Piardi T., Memeo R., Zuccalà V., Luposella M., Patruno R., Zizzo N., Gadaleta P. (2017). The Density of Mast Cells c-Kit^+^ and Tryptase^+^ correlates with each other and with Angiogenesis in Pancreatic Cancer Patients. Oncotarget.

[66] Erba F., Fiorucci L., Pascarella S., Menegatti E., Ascenzi P., Ascoli F. (2001). Selective inhibition of human mast cell tryptase by gabexate mesylate, an antiproteinase drug. Biochem. Pharmacol..

[67] Mori S., Itoh Y., Shinohata R., Sendo T., Oishi R., Nishibori M. (2003). Nafamostat mesilate is an extremely potent inhibitor of human tryptase. J. Pharmacol. Sci..

[68] Humbert M., Castéran N., Letard S., Hanssens K., Iovanna J., Finetti P., Bertucci F., Bader T., Mansfield C.D., Moussy A. (2010). Masitinib combined with standard gemcitabine chemotherapy: In vitro and in vivo studies in human pancreatic tumour cell lines and ectopic mouse model. PLoS ONE.

[69] Marech I., Patruno R., Zizzo N., Gadaleta C., Introna M., Zito A.F., Gadaleta C.D., Ranieri G. (2013). Masitinib (AB1010), from canine tumour model to human clinical development: Where we are?. Crit. Rev. Oncol. Hematol..

[70] Deplanque G., Demarchi M., Hebbar M., Flynn P., Melichar B., Atkins J., Nowara E., Moyé L., Piquemal D., Ritter D. (2015). A randomized, placebo-controlled phase III trial of masitinib plus gemcitabine in the treatment of advanced pancreatic cancer. Ann. Oncol..

[71] Washington K. (2010). 7th Edition of the AJCC Cancer Staging Manual: Stomach. Ann. Surg. Oncol..

[72] Liu X., Cai H., Shi Y., Wang Y. (2012). Prognsotic factors in patients with node-negative gastric cancer: A single center experience from China. J. Gastrointest. Surg..

[73] Sjo O.H., Merok M.A., Svindland A., Nesbakken A. (2012). Prognostic impact of lymph node harvest and lymph node ratio in patients with colon cancer. Dis. Colon Rectum.

[74] Tamura S., Takeno A., Miki H. (2011). Lymph node dissection in curative gastrectomy for advanced gastric cancer. Int. J. Surg. Oncol..

[75] Ranieri G., Grammatica L., Patruno R., Zito A.F., Valerio P., Iacobellis S., Gadaleta C., Gasparini G., Ribatti D. (2007). A possible role of thymidine phosphorylase expression and 5-fluorouracil increased sensitivity in oropharyngeal cancer patients. J. Cell. Mol. Med..

